# Supplementary material for: Rosmarinic Acid Production from Origanum dictamnus L. Root Liquid Cultures In Vitro
Source: Plants (Basel). 2023 Jan 8;12(2):299. doi: 10.3390/plants12020299 (PMC9864065; doi:10.3390/plants12020299)
Supplement: Supplementary file 1 [file plants-12-00299-s001.zip › plants-2084836-supplementary.pdf]

Rosmarinic acid production from *Origanum dictamnus* L. root liquid cultures *in vitro*

Virginia Saropoulou, Charikleia Paloukopoulou, Anastasia Karioti, Eleni Maloupa, Katerina Grigoriadou*

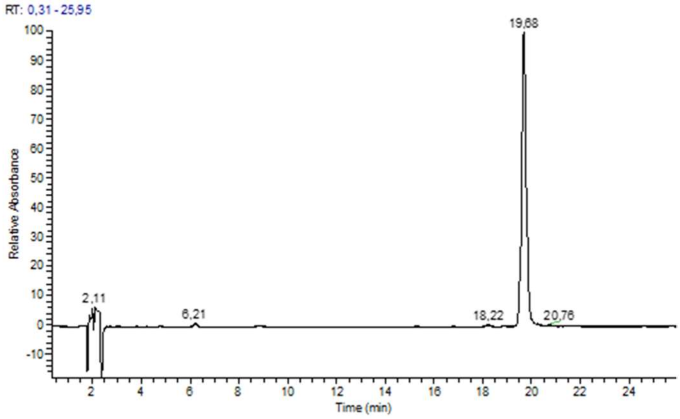

Figure S1 Representative chromatograms of the *Origanum dictamnus* roots at 330 nm. Experimental conditions: column: Zorbax SbAq RP-C18 (150 x 3.0 mm), particle size of 5μm (Agilent) at 30 °C.

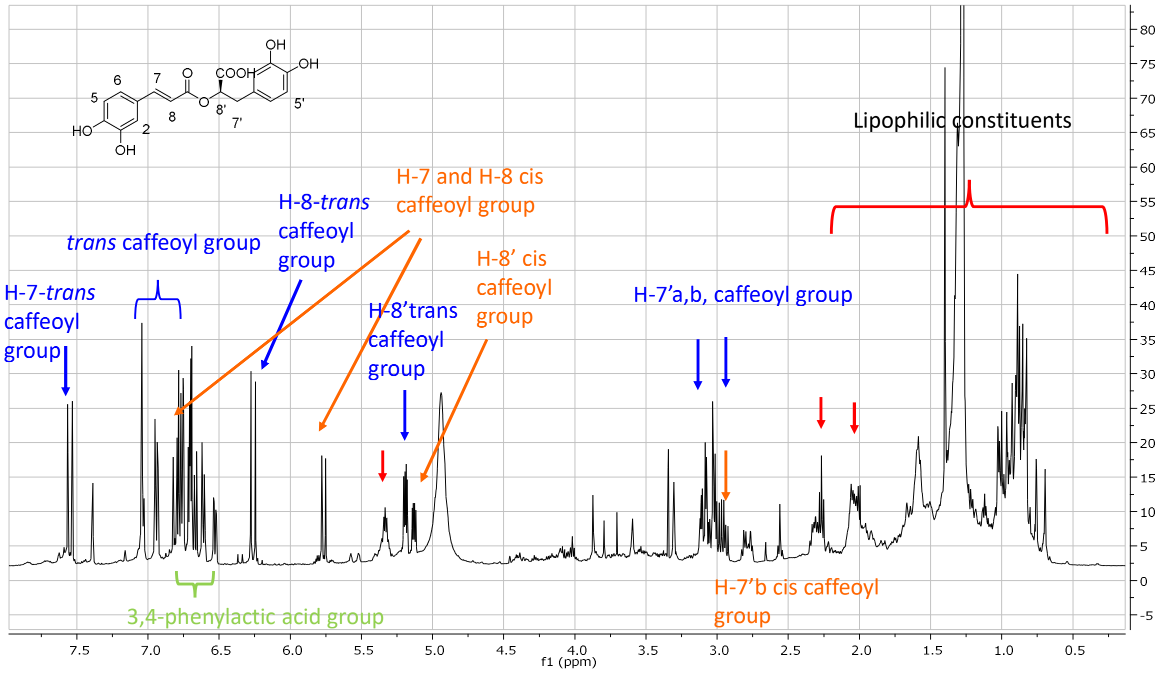

Figure S2. NMR spectrum of a leaves callus extract. In the ^{1}H-NMR spectrum the signals of two ABX groups are observed belonging to two phenolic rings. Protons of the caffeoyl group are shifted downfield: at δ 7.04 proton H-2 appears as doublet with J=1.8Hz due to the meta coupling with proton H-6. Proton H-5 appears as doublet at δ 6.77 with ortho- coupling (J=8.0 Hz) to proton H-6. Proton H-6 appears at δ 6.94 as double-doublet due to coupling to protons H-2 and H-5. Similar signals are observed for the second ABX system belonging to the 3,4-dihydroxyphenylactic acid: H-2′ (δ 6.75, d, J = 1.8Hz), H-5′ (δ 6.70, d, J = 7.8Hz) and H-6′ (δ 6.61, dd, J = 8.2, 2.0). Diagnostics are the signals of the protons H-7 and H-8 of the *trans* (*E*) double bond of the caffeoyl group which appear as two doublets with large coupling constants (J = 15.9 Hz) at δ 7.54 and 6.26, respectively. At δ 5.19 a double doublet appears (J=3.9, 8.6), which corresponds to H-8′ of the methine and is due to the couplings with protons H-7′and H-7′b. These protons are observed as double doublets at δ 3.09 (H-7′a / J= 3.9, 14.1) και δ 2.98 (H-7′b/ J= 8.2, 14.1). In the same spectrum a second series of minor peaks appears due to the isomerization of rosmarinic acid to cis rosmarinic acid. Peaks at δ 6.81 and 5.77 (J = 2.8 Hz) are attributed to protons H-7 and H-8 of the cis rosmarinic acid. Similarly, a double doublet at δ 5.13 is attributed to H-8′ of the cis rosmarinic acid.

In red color, all signals belonging to lipophilic constituents.

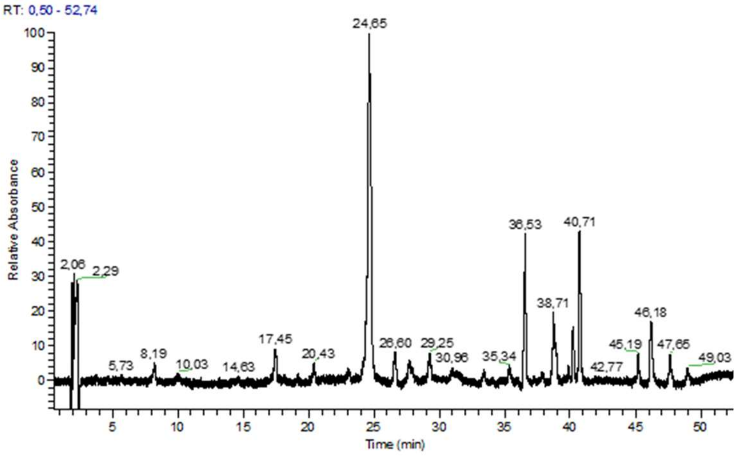

Figure S3. Representative chromatograms of the *Origanum dictamnus* leaves at 330 nm. Peak at 24.65 minutes belongs to rosmarinic acid. Differences in the retention time with figure S1 is due to differences in the elution program

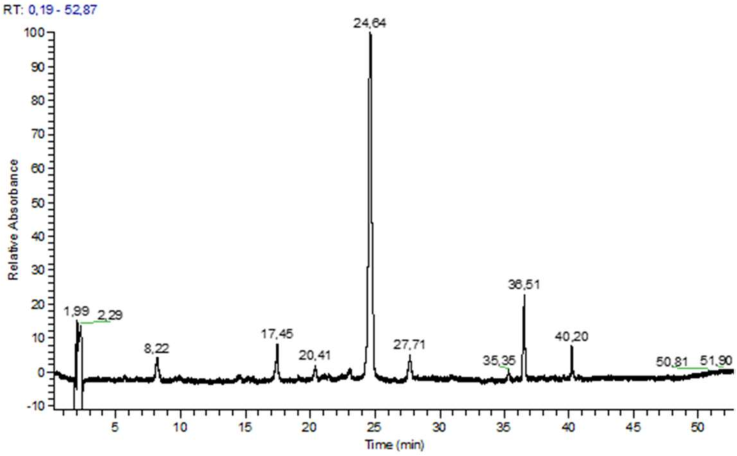

Figure S4. Representative chromatograms of the *Origanum dictamnus* petioles at 330 nm. Peak at 24.65 minutes belongs to rosmarinic acid. Differences in the retention time with figure S1 is due to differences in the elution program.

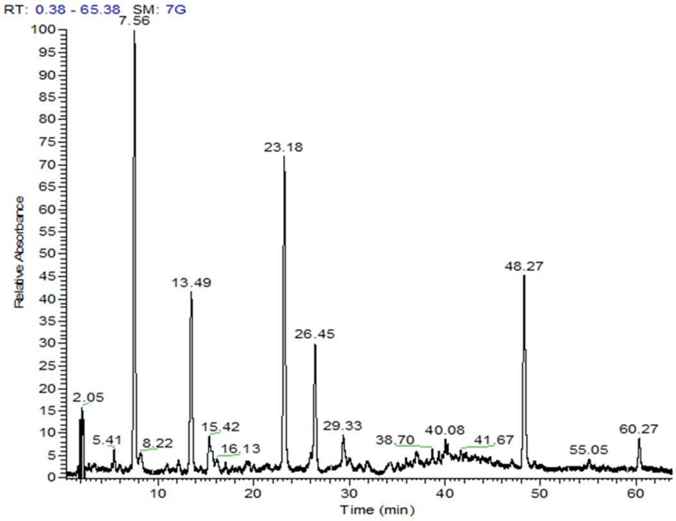

Figure S5. Representative chromatograms of the *Origanum dictamnus* roots at 330 nm. Peak at 23.18 minutes belongs to rosmarinic acid. Differences in the retention time with figure S1 is due to differences in the elution program.
